# A Portable Measurement System Based on Nanomembranes for Pollutant Detection in Water

**DOI:** 10.3390/s25216557

**Published:** 2025-10-24

**Authors:** Luca Tari, Maria Cojocari, Gabriele Cavaliere, Sarah Sibilia, Francesco Siconolfi, Georgy Fedorov, Luigi Ferrigno, Polina Kuzhir, Antonio Maffucci

**Affiliations:** 1 Department of Electrical and Information Engineering, University of Cassino and Southern Lazio, 03043 Cassino, Italy or gcavaliere@unisa.it (G.C.); sarah.sibilia@unicas.it (S.S.); francesco.siconolfi@unicas.it (F.S.); ferrigno@unicas.it (L.F.); 2 EUT+ Institute of Nanomaterials and Nanotechnologies—EUTINN, European University of Technology, European Union, 03043 Cassino, Italy; maffucci@unicas.it; 3 Department of Physics and Mathematics, Center for Photonics Sciences, University of Eastern Finland, 80100 Joensuu, Finland; maria.cojocari@uef.fi (M.C.); georgy.fedorov@uef.fi (G.F.); polina.kuzhir@uef.fi (P.K.); 4 Department of Information and Electrical Engineering and Applied Mathematics, University of Salerno, 84084 Fisciano, Italy; 5 E-lectra srl, 03043 Cassino, Italy

**Keywords:** electrical impedance spectroscopy, environmental monitoring, nanomembranes, nanotechnology, real-time sensing, smart monitoring

## Abstract

This work presents the design, the development and the experimental validation of a portable, low-cost sensing system for the detection of waterborne pollutants. The proposed system is based on Electrochemical Impedance Spectroscopy and PPF+Ni nanomembrane sensors. Designed in response to the increasing demand for in situ water quality monitoring, the system integrates a simplified, scalable EIS acquisition architecture compatible with microcontroller-based platforms. The sensing configuration utilises the voltage divider principle, ensuring simplicity in signal conditioning by allowing compatibility with different electrode types through passive impedance matching. In addition, new merit figures have been proposed and implemented to analyse the measures. The proposed platform was experimentally characterised for its measurement stability, accuracy and environmental robustness. Sensitivity tests using benzoquinone as a target analyte demonstrated the capability of detecting concentrations as low as 0.1 mM with a monotonic response over increasing concentrations. A comparative study with a commercial electrochemical system (PalmSens4) under identical conditions highlighted the higher resolution and practical advantages of the proposed method despite operating with a lower impedance range. Additionally, the system exhibited reliable discrimination across tested concentrations and greater adaptability for integration into field-deployable environmental monitoring platforms. Future developments will focus on optimising selectivity through new sensor materials and analytical modelling of uncertainty propagation in the analysis based on defined figures of merit.

## 1. Introduction

The rapid pace of over-industrialisation in recent years has led to the generation of vast quantities of industrial waste, significantly exacerbating environmental pollution. Water contamination has emerged as a significant concern among the resulting issues due to its severe implications for human health and ecological balance. As a result, water quality monitoring and remediation have been identified as priority objectives within the framework of the United Nations’ 2030 Agenda for Sustainable Development [1]. Despite the availability of several efficient systems for water monitoring [2,3,4,5,6,7], there remains a pressing need for novel and reliable technological solutions that can effectively meet three critical criteria: real-time monitoring, in situ detection, and low-cost instrumentation. The primary challenges lie in the current limitations of low-cost sensing elements and portable sensing platforms [8,9,10,11,12,13,14]. From a methodological standpoint, the most accurate detection techniques are traditionally associated with established analytical methods such as chromatography (e.g., High-Performance Liquid Chromatography, HPLC [15]) and mass spectrometry [16]. While these approaches are highly reliable, they are inherently expensive, time-consuming and labour-intensive and are typically confined to laboratory settings, limiting their applicability for on-site analysis. Conversely, techniques suitable for in situ and real-time applications include spectrophotometry [17], EIS [18,19], electrochemical methods such as voltammetry [20,21,22], and emerging approaches based on plasmonic resonances [23]. On the materials front, recent progress in the synthesis and engineering of nanomaterials has introduced a wide array of promising alternatives, owing to their exceptional sensitivity and scalability [24,25]. Within this context, nanomembranes have garnered significant attention. Their two-dimensional geometries offer enhanced surface-to-volume ratios, improving sensor performance [25,26]. The critical role of surface interactions is also well documented in traditional materials such as gold [27]. Graphene-based nanomembranes, in particular, exhibit high sensitivity to non-specific environmental factors such as pH, ionic strength, and temperature. Consequently, selective detection of target pollutants typically requires functionalisation with specific molecular receptors [28]. Furthermore, the integration of Machine Learning techniques has been explored as an alternative strategy to improve selectivity [21].

This work builds upon a previous study by the authors [18,29,30], which demonstrated the feasibility of using nanomembranes composed of PPF, pyrolytic carbon, and multilayer graphene for EIS-based detection of waterborne pollutants. The laboratory results, obtained with high-precision benchtop instruments, showed considerable promise. However, further development is necessary to enhance sensitivity and detection capabilities, particularly at very low pollutant concentrations. Continuing the research of the authors, the present study pursues two primary objectives. First, it aims to design and develop a dedicated measurement system, based on EIS, specifically tailored to integrate the previously investigated nanomembranes. This system is conceived to be automatic, low cost, and portable. Second, the study introduces novel EIS-based figures of merit for post-processing to improve the overall performance of the sensing platform, encompassing both the nanomembrane and the measurement system. To achieve these goals, the following steps have been undertaken:

(i) Development and characterisation of an automatic and portable EIS-based measurement system, offering competitive sensitivity and repeatability relative to high-performance benchtop instruments;
(ii) Integration of low-cost nanomembranes into the architecture of the portable device and characterisation of their sensing performance for pollutant detection;
(iii) Design of novel measurement protocol and analysis method to enhance sensitivity and repeatability;
(iv) Comparison with certified reference commercial instruments, focusing on its ability to detect low concentrations of pollutants.

The pollutant chosen to test the proposed system is benzoquinone, as it serves as a representative example of toxic compounds released through industrial and anthropogenic activities that pose serious public health risks. Prolonged exposure to BQ is known to cause irritation, mutagenesis, and acute poisoning [31].

The article is structured as follows: Section 2 describes the fabrication and structural characteristics of nanomaterials used as sensors and introduces the operational principles of applied EIS. Section 3 presents the proposed portable measurement system, discusses its metrological characterisation, and defines the experimental procedures. Measurement results are presented and analysed in Section 4, including the introduction of novel figures of merit for electrochemical analysis of chemical species. Section 5 compares the developed system with a commercial instrument. Finally, Section 6 provides the conclusions of the study.

## 2. Background

### 2.1. Nanomembrane Synthesis and Applicability as Sensing Devices

The nanomembranes employed in this work were fabricated from PPF deposited on substrates pre-coated with a Ni catalyst layer, a configuration previously validated by the authors for its sensing performance in comparison with other nanomaterials [18]. This structure, called PPF+Ni, results in a well-ordered graphitic film characterised by an electronic band structure similar to that of graphite and graphene while maintaining a relatively simple and scalable fabrication process. Their synthesis process is summarised here (additional details can be found in references [32,33]). To investigate the influence of the catalyst and substrate on film properties, different Ni layer thicknesses (3 nm, 10 nm, and 30 nm) were sprayed onto various dielectric substrates, including silicon nitride (Si_3_N_4_), silicon dioxide (SiO_2_) on silicon wafers, and fused silica. A 300 μL volume of AZ nLoF photoresist (1:4) was spin-coated at 3000 rpm for 60 s and subsequently baked at 110 °C for one minute, yielding a resist film approximately 230 nm thick. The samples were then subjected to pyrolysis under a hydrogen atmosphere (5 sccm, 0.1 mBar) at 800 °C for 10 min, followed by passive overnight cooling in static hydrogen. The final thickness of the resulting nanomembranes was measured to be approximately 60 nm. It should be noted that the amount of Ni involved is extremely small and therefore its concentration is negligible such that it will not lead to contamination if the sensor is used in open water [32,33].

**Figure 1:**
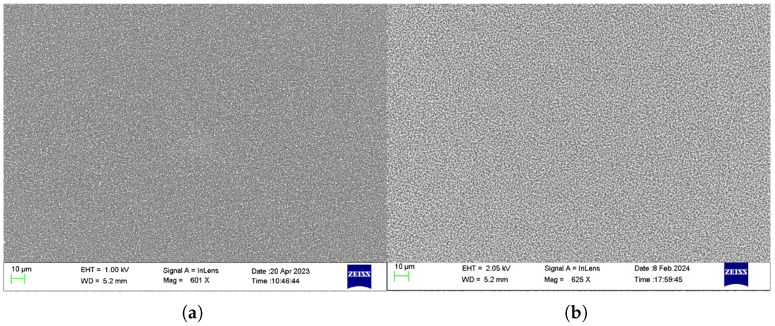
SEM images of two graphitic films after annealing: (**a**) 10 nm Ni layer, FS substrate; (**b**) 30 nm Ni layer, FS substrate.

**Figure 2:**
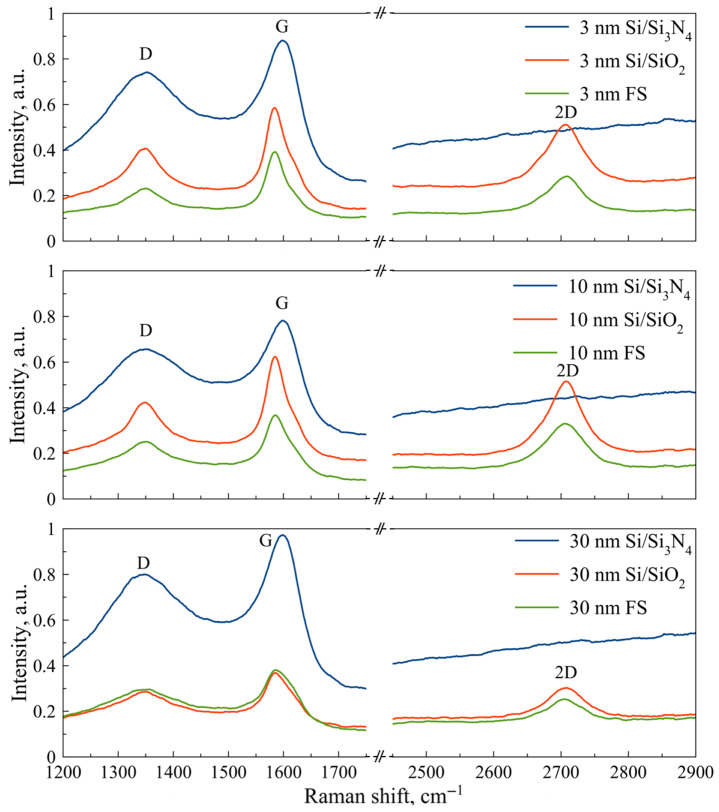
Raman spectra of the graphitic film for 3 nm, 10 nm, and 30 nm Ni layer on Si/Si_3_N_4_, Si/SiO_2_, and FS substrates.

Structural characterisation was performed to assess the uniformity and crystallinity of the graphitic films. SEM imaging (Zeiss LEO 1550) revealed continuous films with submicron Ni island formations, which were observed to increase in density with thicker catalyst layers, as shown in Figure 1. Raman spectroscopy conducted using a Renishaw inVia spectrometer (514 nm laser, 2400 lines/mm grating, 50 × objective), confirmed the presence of D and G peaks in all samples. Notably, significant 2D peaks—indicative of crystalline graphitic structures—were detected only in samples fabricated on Si/SiO_2_ and fused silica substrates, according to Figure 2 [34]. Based on these results, fused silica (FS) was selected as the preferred substrate for reliable conductivity measurements. A catalyst thickness of 10 nm was chosen as optimal, striking a balance between uniformity and 2D peak intensity. Sheet resistance was measured at approximately 200 Ω/sq using the Van der Pauw method with a Signatone probe station and a Gwinstek multimeter.

### 2.2. Operating Principle: Electrochemical Impedance Spectroscopy

The detection mechanism adopted in this work is based on EIS, a technique selected for its compatibility with low-cost, miniaturised sensor systems and real-time, in situ monitoring applications.

Prior research has shown that adsorption of external chemical species onto the used nanostructured materials for this work produces measurable modifications of their electrochemical and electrical properties, which can be robustly captured through impedance measurements [35,36,37,38,39]. Building on the authors previous works [18,29], the present sensor exploits a two-dimensional nanomembrane whose exceptionally high surface-to-volume ratio provides a high capability of adsorption, thereby amplifying the transduction of molecular interactions into changes in the electrical impedance response: external molecules perturb the crystalline lattice of the membrane (through physisorption or chemisorption), leading to permanent deformations. These perturbations modulate charge transport by altering carrier scattering and the effective number of conducting channels, which in turn modifies the material conductivity and related impedance parameters, according to the generalized Ohm’s law at the nanoscale [40]. EIS is used to detect these changes in the nanomembrane impedance, whose frequency-dependent variations are related to the nature and the intensity of molecular adsorption on the sensing surface. Infact, as described in [40], the conductivity of the material is strongly dependent on frequency.

**Figure 3:**
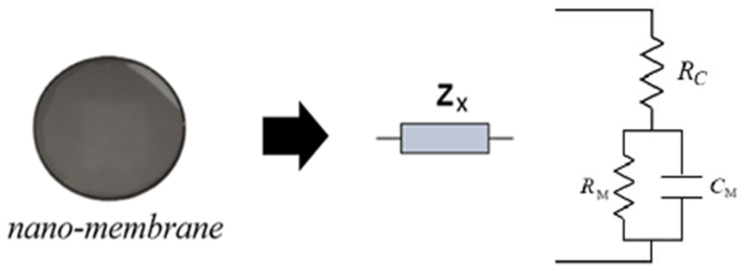
The EIS measures the electrical impedance of the nanomembrane, shown on the left, according to the circuit model shown (${\dot{Z}}_{x}$ represents the nanomembrane impedance).

**Figure 4:**
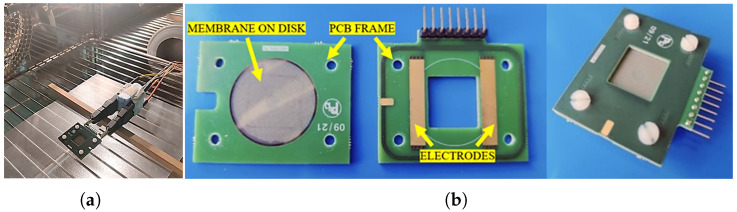
(**a**) shows the sensor placed inside the climatic chamber connected to the impedance meter during the characterisation phase. (**b**) shows in detail the PCB support used for interfacing with the nanomembrane.

Furthermore, preliminary experimental observations established that the most diagnostic features appear in the impedance modulus, with comparatively minor changes in the phase. For this reason, in the following sections, only the impedance module will be considered. Therefore, EIS is particularly well suited for this architecture owing to its high capability to detect crystalline lattice ideformations in the nanomembranes, scalability across frequency and device dimensions and potential straightforward future integration within microcontroller-based instrumentation. In this context, the authors have mastered this technology thanks to previous applications of EIS in different fields of application to monitor the health of water using graphene disks [29,30,41,42], to diagnose the presence of pollutants in hydraulic fluids [43,44] and for diagnostics in the automotive [45] and aerospace fields [46]. Consistent with those findings, the nanomembrane’s behaviour under EIS can be modelled with an equivalent electrical circuit. As shown in Figure 3, the model consists of a parallel $R_{M}-C_{M}$ branch representing the membrane’s resistive and capacitive behaviour, in series with a contact resistance $R_{C}$. In addition, Figure 3 (and Figure 4) shows the nanomembrane in question. Future work will investigate the correlation between variations in these circuit parameters and the presence of specific pollutants.

## 3. Proposed Automatic Portable Measurement System

A comprehensive design and experimental characterisation process was conducted to realise a portable instrument capable of performing EIS measurements for pollutant detection. This included the preliminary characterisation of the sensor, the development of the analogue front-end, the selection of the acquisition and analogue-to-digital conversion system based on noise and sensitivity requirements and the validation of metrological performance through targeted experimental testing. This approach ensured that both the design and implementation phases were aligned with the final performance objectives of the sensor system.

### 3.1. Environmental Characterisation of the Nanomembranes

The first step involved the experimental characterisation of the sensors in question, i.e., the nanomembranes, in terms of impedance. The aim was to assess the stability and repeatability of the sensors by analysing the frequency response under conditions of real environmental variability. To this end, experiments were carried out on five membranes in the presence of controlled variations in temperature and humidity. Two test protocols were as follows:

- Temperature variation: Measurements were conducted at −40 °C, −20 °C, 0 °C, 20 °C, and 40 °C, under constant humidity (10% for temperatures higher than 0 °C, and 0% for the other ones, as it is not possible to reach it);
- Humidity variation: Measurements were conducted at 10%, 25%, 50%, 75%, and 90% relative humidity, under constant temperature (20 °C).

**Figure 5:**
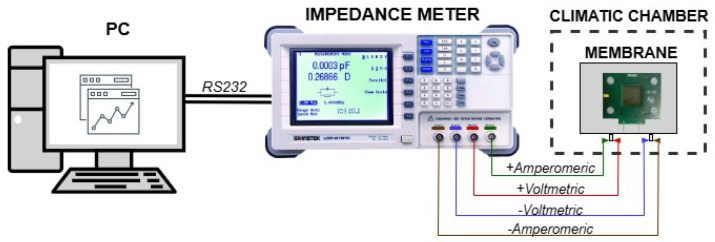
Measurement setup used for characterising nanomembranes. It consists of a sensor embedded in the PCB support, an impedance meter and a PC to manage operations via RS232 communication.

Each test was conducted by placing the membranes in an ACS DY110 climatic chamber (Angelantoni, Massa Martana, Italy) and performing a spectroscopic analysis with a GW Instek LCR1920 impedance meter (Good Will Instrument Co., Taipei, Taiwan). Figure 5 shows the setup realised for the characterisation phase.

The frequency range considered was between 20 Hz and 1 MHz with logarithmic steps, for a total of 15 frequencies analysed. The response was represented according to a “Modulus-Phase” impedance model. Characterisation was performed on five membranes. Figure 4a shows the nanosensor placed inside the climatic chamber and connected to the measuring instrument. For optimal connectivity with the measuring instrument and signal stability, the membrane was mounted onto a custom PCB shown in Figure 4b, including a grounded guard ring to minimise parasitic capacitance.

**Figure 6:**
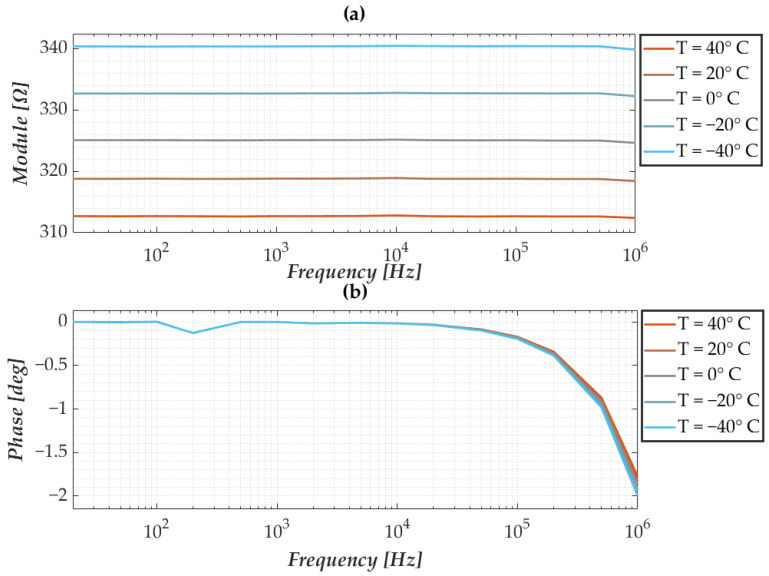
Characterisation at varying temperatures and fixed humidity showing (**a**) the modulus and (**b**) the phase of the nanomembrane’s impedance.

**Figure 7:**
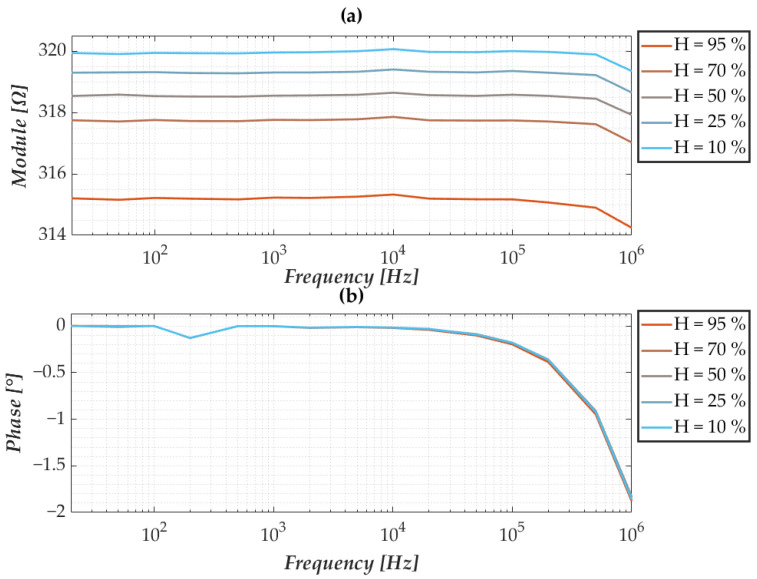
Characterisation at varying humidity and fixed temperature showing (**a**) the modulus and (**b**) the phase of the nanomembrane’s impedance.

As mentioned in the previous section, the sensors in question are manufactured by an industrial process that results in variability in terms of the nominal impedance of the nanomembrane itself. However, as also highlighted in the authors’ previous research, despite the discrepancies in terms of impedance, the frequency behaviour is the same between different nanomembranes. In this regard, Figure 6 and Figure 7 show the response of a membrane taken as a reference when environmental conditions vary. The modulus and phase responses show excellent frequency stability when temperature and humidity vary, except for a slight decrease at higher frequencies. Furthermore, considering the various test conditions, there appears to be an increase in the impedance modulus when the temperature decreases, while the phase does not vary. The same considerations can be made when observing the responses to changes in humidity, whereas humidity increases, and there is a decrease in the impedance modulus and, again, no change in the phase response.

From the results just discussed, given the frequency stability and the reduced variability due to environmental factors (under real test conditions, variations are minimal during a test as they are assumed not to be for continuous monitoring), it was decided not to implement future compensations in the post-processing phase.

### 3.2. Development of the Transduction Circuit

**Figure 8:**
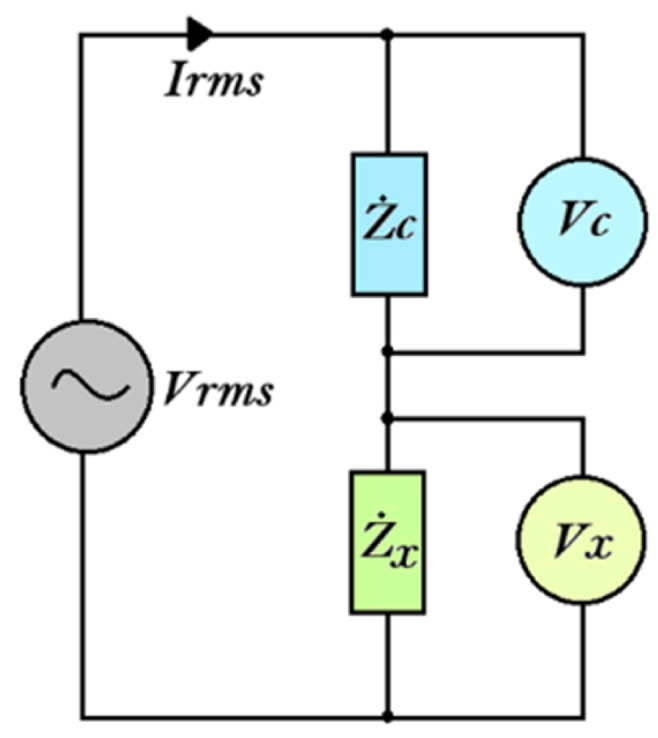
Measurement circuit used to implement the impedance spectroscopy: “${\dot{Z}}_{c}$” represents the sample impedance, whereas “${\dot{Z}}_{x}$” represents the unknown impedance to be measured.

The transduction circuit was designed considering the voltage limits of the acquisition system and the bandwidth requirements necessary for EIS operation. As determined in prior work [18], the nanomembrane must be excited and its current response measured across a frequency spectrum ranging from 20 Hz to 1 MHz, with a current limit not exceeding approximately 20 mA. Based on this, a voltage divider configuration was selected for current measurement. This measurement configuration is reported in Figure 8, where ${\dot{Z}}_{c}$ represents the sample impedance and ${\dot{Z}}_{x}$ represents the unknown impedance to be measured.

The impedance ${\dot{Z}}_{c}$ was set to 50 Ω using a precision 0.1% BNC RF termination model TRM-2048-MC-BNC-10 (TRM Microwave, Bedford, MA, USA) with high frequency stability up to 1 GHz, chosen to ensure reliable operation up to 1 MHz and to match the expected order of magnitude of the nanomembrane impedance. This value was selected based on a preliminary characterisation discussed in the previous subsection to assess the nominal range of the impedance module. The range obtained from the characterisation was less than 500 Ω. According to this measurement method, the following equation can be defined as follows:

(1) $${\bar{V}}_{g}={\bar{V}}_{c}+{\bar{V}}_{x}$$

(2) $${\dot{Z}}_{x}=\frac{{\bar{V}}_{x}}{{\bar{I}}_{x}}$$

(3) $${\bar{I}}_{x}=\frac{{\bar{V}}_{c}}{{\dot{Z}}_{c}}$$

(4) $${\dot{Z}}_{x}=\frac{{\bar{V}}_{x}}{{\bar{V}}_{c}}{\dot{Z}}_{c}$$

where ${\bar{V}}_{g}$ represents the AC generated voltage equal to the sum of the voltage drop on the sample impedance ${\bar{V}}_{c}$ and the voltage drop on the unknown impedance ${\bar{V}}_{x}$, while ${\bar{I}}_{x}$ represents the current of the measurement circuit.

To get an idea, we applied 0.5 VRMS to the circuit.

- Considering a nanomembrane impedance equal to 50 Ω (best condition for the voltage divider), the resulting current amplitude is 5 mA, with corresponding voltage drops of 0.25 V ($V_{x}$) and 0.25 V ($V_{c}$);
- Considering a nanomembrane impedance equal to 500 Ω (worst condition for the voltage divider), the resulting current amplitude is around 0.909 mA, with corresponding voltage drops of 0.4545 V ($V_{x}$) and 0.0455 V ($V_{c}$).

These values allow a good measurement range for operating ADC devices in each case. Furthermore, the chosen solution simplifies the implementation of the system by avoiding the use of adaptive references.

### 3.3. The Choice of the ADC Board

**Figure 9:**
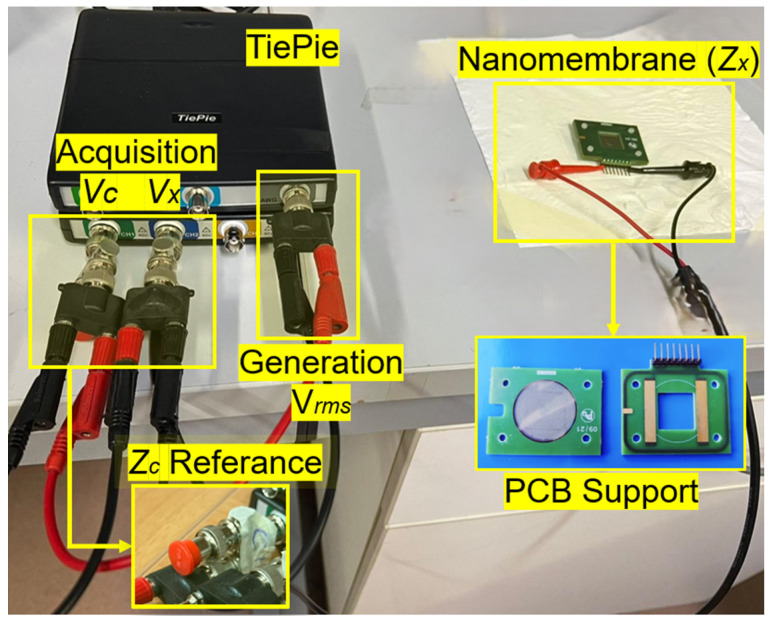
Setup realised for the experimental measurement campaign.

A synchronised dual-channel acquisition system was implemented to generate sinusoidal excitation signals and simultaneously acquire voltage responses across the reference and the membrane. This has been made using two programmable TiePie oscilloscopes: one for differential acquisition using a TiePie HS6 [47] and the other for generation using a TiePie HS5 [48], as shown in Figure 9.

Control and data processing were managed through a custom MATLAB^TM^ (R2024b version) application. The system thus realised in its current proof-of-concept state is less expensive compared to complex laboratory instruments, portable and easily scalable to prototype hardware by increasing the Technology Readiness Level. Once the nanomembrane (${\dot{Z}}_{x}$) and reference (${\dot{Z}}_{c}$) impedances were connected, the system applied a frequency-variable sinusoidal excitation, measuring both $V_{x}$ and $V_{c}$ to calculate ${\dot{Z}}_{x}$ (modulus and phase) across the entire frequency range of interest. The required ADC resolution was derived from the precision of ${\dot{Z}}_{c}$ (0.1%) using the formula

(5) $$N_{bit}=\frac{1}{2}\log_{2}\left(\frac{V_{eff}}{N_{eff}}\right)-1.774$$

where

- $N_{bit}$ is the number of bits of ADC;
- $V_{eff}$ is the RMS value of the signal;
- $N_{eff}$ is the RMS value of the signal noise.

This yielded a minimum resolution of 10-bit, well within the 14-bit resolution provided by the selected system. This choice optimizes between the required measurement resolutions and the effect of the voltage reference instability on the ADC. As mentioned before, for optimal connectivity and signal stability, the membrane was mounted onto a custom PCB support, including a grounded guard ring to minimise parasitic capacitance. The used metrological specifications are shown in Table 1; these were also defined thanks to the characterisation process of the experimental setup realised (Section 3.4) to guarantee the containment of the measurement error within certain limits; a wide range of exploration in terms of frequency, reduced measurement times.

### 3.4. Experimental Characterisation of the Developed Instrument

**Figure 10:**
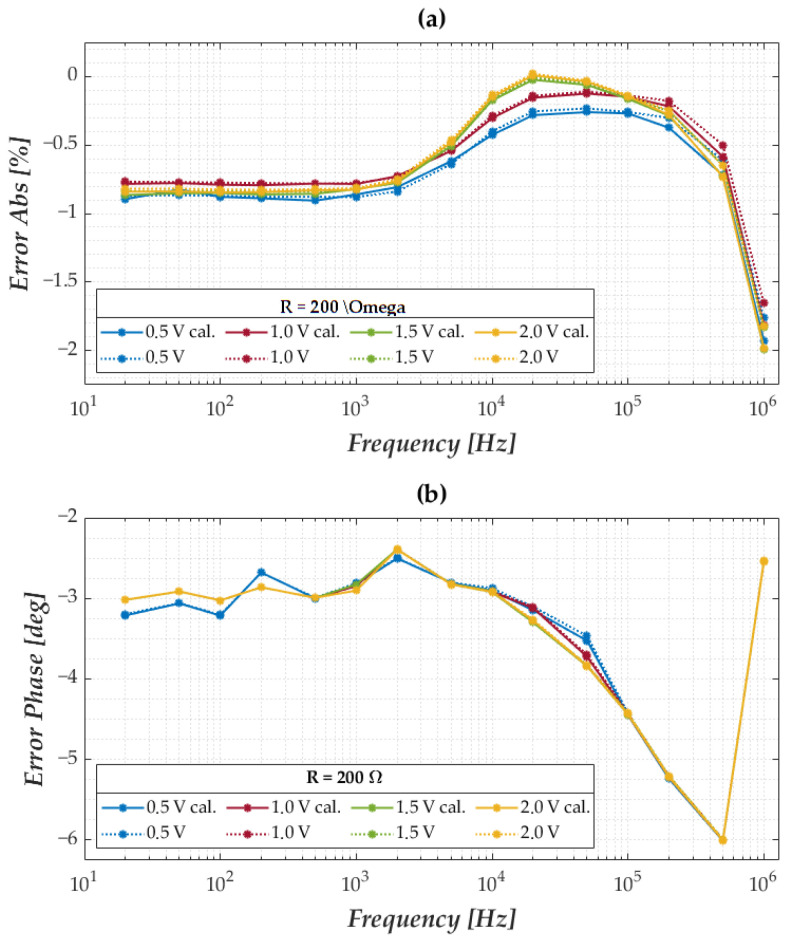
Trend of the experimental errors on the impedance modulus (**a**) and phase (**b**) compared to the reference instrument for all tested forcing voltages at a test impedance value of 200 Ω.

An extensive performance characterisation of the entire measurement chain was conducted to assess the accuracy and repeatability of the measurement system under different excitation amplitudes and test impedances. This made it possible to estimate possible variations in system performance due to impedance variability of different membranes. In particular, impedance values of 50 Ω, 100 Ω, 200 Ω and 500 Ω have been tested according to the nanomembrane module nominal range. The four impedance values tested were obtained using laboratory sample resistors. The decision to use only resistors is justified by the nature of the membrane, as highlighted by the experimental characterisation shown in Figure 6b and Figure 7b, i.e., almost purely ohmic, with the phase tending towards zero. With regard to excitation voltages, four different voltage levels were tested, equal to 0.5 V, 1 V, 1.5 V and 2 V, spanning a frequency range between 20 Hz and 1 MHz. The maximum voltage amplitude $V_{g}$ was set according to the maximum current supportable ($I_{x}$ = 20 mA) in the critical test condition ($Z_{x}$ = $Z_{c}$ = 50 Ω). For each combination, 30 repeated measurements were taken and compared to results obtained with the LRC1920 meter. Figure 10 presents the measurement error trends for modulus (a) and phase (b) at a test impedance of 200 Ω (intermediate condition of the tested range). A stable error in modulus is observed up to tens of kHz, with a minor bell-shaped deviation appearing near 1 MHz. The maximum modulus error remains below 2% across all conditions. Phase errors are similarly modest, staying below 1% up to 10 kHz, with larger deviations beyond. It is evident from the performance obtained that the system does not require any additional hardware and/or software compensation.

The summarised performance metrics are reported in Table 2 and Table 3. As expected, larger discrepancies between ${\dot{Z}}_{x}$ and ${\dot{Z}}_{c}$ increase measurement error. Among all tested configurations, the 0.5 V excitation consistently produced the lowest average and peak errors, indicating it as the most stable and accurate operating condition.

## 4. Experimental Protocols and Results Discussion

**Figure 11:**
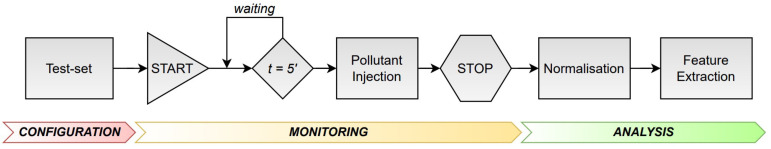
Flowchart used during the monitoring campaign and analysis of the results obtained. It illustrates the three operational steps carried out: configuration, monitoring and analysis.

This section describes in detail the monitoring campaign conducted and the analysis of the results obtained. To facilitate the understanding of the reader, Figure 11 shows a flowchart illustrating the main operational phases. The overall activity is divided into three main steps: configuration, monitoring and analysis.

In the first phase, the measurement system developed is configured according to the type and methods of testing envisaged. Details of the test protocol and operating methods adopted are given in Section 4.1. This is followed by the monitoring phase, during which experimental data are acquired using the EIS technique. At the end of the monitoring campaign, the collected data are analysed. As described in Section 4.2, this phase includes the definition and implementation of new parameters and evaluation features useful for investigating the response characteristics of nanomembranes. For completeness, the response curves of the membranes under different test conditions are also presented and discussed qualitatively in order to provide a clearer picture of the observed behaviour.

Finally, the quantitative results of the process are reported in Section 4.3, where the measured output variations and response times are analysed.

### 4.1. Measurement Protocols and Case Studies Output

A dedicated experimental campaign was conducted, integrating the nanostructured membrane with the previously described portable acquisition system, to assess its sensing capabilities when exposed to controlled quantities of a selected pollutant. For methodological consistency, each test involved the exposure of a newly prepared membrane to aqueous solutions containing increasing concentrations of a single chemical species. The pollutant employed in this study was *para*-benzoquinone (p-benzoquinone, 1,4-benzoquinone; chemical formula C_6_H_4_O_2_), a prototypical member of the quinone family and a conjugated diketone that acts as a strong electron acceptor/oxidant and is frequently encountered as a hazardous by-product of diverse industrial processes. For all experiments, p-benzoquinone was dissolved in a 0.1 M acetate buffer at pH 4, supplemented with 0.1 M KCl.

The chemical environment used in this work was intentionally chosen to reflect the current, non-functionalised state of graphite-based nanomembranes. This strategic design choice allowed the authors to stabilise the basic transduction physics, refine the measurement protocol, and validate the entire signal acquisition and processing chain on a low-cost, portable hardware platform before introducing application-specific chemical functionalisation operations on the nanosensor. At this stage of the work, the aim is not yet to evaluate the selectivity of nanomaterials to pollutants, but rather to mature the platform and analysis methodology by rigorously evaluating the basic metrological performance under controlled conditions, thus reducing risks in subsequent development phases. Therefore, since the membranes are not yet functionalised, experiments have not been performed on real water matrices, which would have been premature and confused by non-specific adsorption of the chosen pollutant due to more complex matrices; consequently, the present work is limited to simplified buffer conditions appropriate to the current level of technological maturity.

Four concentration levels (0, 0.1, 1 and 10 mM) were tested. Although data on actual BQ concentrations in industrial effluents are scarce—being an organic pollutant emerging from aromatic oxidation processes—the range chosen reflects that commonly used in experimental studies. The upper limit of 10 mM, probably beyond real-world conditions, was selected as the limiting case to verify the performance of the nanosensor and ensure its characterisation under both realistic and extreme conditions.

In detail, the system configuration in accordance with Figure 11 is as follows:

- All tests were performed on PPF+Ni membranes at a controlled room temperature of 20 °C.
- The pollutant concentrations considered were 0 mM, 0.1 mM, 1 mM and 10 mM. These quantities were obtained using a precision balance and a laboratory micro-pipette. The concentration at 0 mM refers to tests conducted without the pollutant.
- Four tests were carried out for each concentration, replacing the membrane used with a new one each time (deformations on the crystal lattice induced by interaction with external molecules are permanent, so they cannot be reused [18,29,30]).
- Each membrane used in a particular test was given an identification number (M#). Table 4 summarises this information.
- The duration of each experiment varied slightly, approximately 20 min. The variability in time is related to the response and minor delays in the system.
- The injection of pollutants via micro-pipette takes place at the 5-min mark, thus defining a baseline. The evolution of the system follows this.
- EIS was performed at logarithmic steps (1, 2, 5, 10, …) in a frequency range of 20 Hz to 1 MHz with a forcing voltage of 0.5 V, according to the previous sections (Table 1).
- The measurement time (time between measurements) is 2 s.
- The estimated impedance has been represented for ease of understanding according to a “Modulus-Phase” model. In particular, the impedance measurements were only analysed in terms of modulus, in line with previous results and previous research [29,30] which indicated that the most relevant information regarding the response of the tested membranes lies in the impedance amplitude.

It should be noted that the number of measurements acquired for each experiment is high. Specifically, considering the duration of the test and the measurement time, approximately 600 EIS measurements were collected for each membrane.

**Figure 12:**
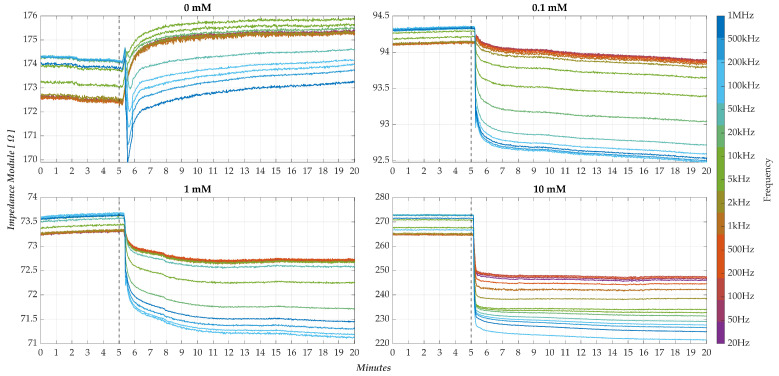
Response curves of the modulus of impedance offered by the nanomembranes at different test conditions (pollutant concentration levels). At the 5′ min, the drop (H_2_O + pollutant) on the nanomembrane occurs.

Figure 12 presents a qualitative overview of the initial results obtained for the four concentration levels tested for four different membranes (M1, M5, M9, M13). The graphs show the trends during the test time of the frequency responses of the impedance modulus. During the baseline, i.e., before the pollutant was injected, the sensor response was recorded as being extremely stable. In other words, the impedance measured for each frequency was almost constant, in line with the characterisation results reported previously in Section 3.1. A clear variation in the behaviour is observed at the pollutant injection at 5 min. It is observed that this variation varies with the concentration level. These preliminary results underline the sensitivity of the membrane to the presence of BQ. However, the limited repeatability observed in the various tests highlights the need for alternative figures of merit to improve the robustness and performance of the system. In particular, as mentioned above, the nanomembrane manufacturing process is such that it does not ensure a uniform nominal impedance between different membranes. This makes it challenging to analyse and compare corresponding responses under various test conditions.

### 4.2. Definition and Implementation of Novel Figures of Merit

In light of the initial findings, a novel methodology proposed in part by the authors in [29,30] for post-processing the sensor output was defined and implemented to enhance both comparability and interpretability of results. Specifically, according to Figure 11, the membrane response is now expressed as a normalised percentage variation of the impedance modulus with respect to the baseline condition, as a function of time and frequency.

This approach is mathematically formalised in Equation (6).

(6) $$R_{m}\left(f_{i},t\right)\;\left[\%\right]=\frac{|{\dot{Z}}_{m}\left(f_{i},t\right)\left|-\right|{\dot{Z}}_{m,B}\left(f_{i}\right)|}{|{\dot{Z}}_{m,B}\left(f_{i}\right)|}\;\cdot \;100\;\;\forall \;f_{i}\in \left[20\;\mathrm{Hz}÷1\;\mathrm{MHz}\right]$$

where

- $R_{m}\left(f_{i},t\right)$ is the membrane response in the time domain at a certain frequency $f_{i}$;
- $|{\dot{Z}}_{m}\left(f_{i},t\right)|$ is the membrane measured impedance module over time at a certain frequency $f_{i}$;
- $|{\dot{Z}}_{m,B}\left(f_{i}\right)|$ is membrane impedance module at the baseline condition at a certain frequency $f_{i}$.

This baseline averaging approach is justified by the high stability of the membrane response in the absence of pollutants and mitigates potential biases that might arise from arbitrary point selection. Given the baseline time (5 min) and the chosen measurement time (2 s), this average is calculated for each frequency tested by considering approximately 150 measurements. The normalisation strategy shifts all curves to a common zero reference, thereby facilitating a direct comparison between different membranes and experimental conditions. Such representation allows for the evaluation of sensor performance irrespective of intrinsic variabilities among individual membranes—variabilities that are inherent to low-cost industrial fabrication processes and which could otherwise compromise the integrity of the analysis.

**Figure 13:**
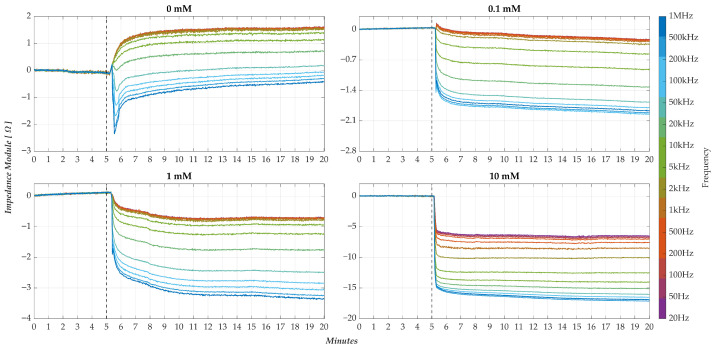
Response curves of the percent variations in impedance modulus, compared to the baseline, offered by the nanomembranes under different test conditions (pollutant concentration levels). At the 5′ min, the drop (H_2_O + pollutant) on the nanomembrane occurs.

To illustrate the effectiveness of this approach, Figure 13 presents the normalised response curves for the same four membranes shown above (M1, M5, M9, M13), each corresponding to one of the pollutant concentrations under investigation. Comparing the responses obtained with the preceding un-normalised condition (Figure 12), the convenience of the proposed approach is evident, especially regarding the amplitude distribution of the responses at the different frequencies.

In particular, for each test condition, one can appreciate a systematic tendency after the drop: high-frequency responses are placed at the bottom and, conversely, low-frequency responses at the top. Furthermore, a detailed analysis of the curves reveals the following.

- In the control condition (0 mM, Figure 13a), low-frequency components (<10 kHz) exhibit a slight positive variation;
- Upon pollutant introduction (Figure 13b,d), the same frequency components exhibit a consistently negative variation, whose magnitude increases with concentration.

As a sign of variation, this inversion provides a qualitative indicator of pollutant presence and concentration. Notably, such directional behaviour highlights the capacity of the sensor, not only for detection but also for preliminary discrimination of concentration levels.

To quantitatively assess this performance, two figures of merit were defined.

1. The normalized percentage response at the minimum measured frequency (20 Hz—with frequency taken as a reference and low frequencies being more significant based on reported outputs);
2. The spectral spread of the response across all frequencies, calculated at each time instant $t_{i}$ using Equation (7):

(7) $$s_{m}\left(t_{i}\right)\;\left[\%\right]=\max\langle R_{m}\left(f,t_{i}\right)\rangle -\min\langle R_{m}\left(f,t_{i}\right)\rangle$$

This metric quantifies the difference between the maximum and minimum normalised response values across all frequencies at a given time, thereby offering a synthetic measure of the spectral distribution’s amplitude.

To provide a quantitative analysis, for each membrane and test condition, both figures of merit were calculated at discrete time intervals after pollutant injection: 30 s, 60 s, 90 s, 120 s, 150 s and 180 s. Following this, the mean values and standard deviation were then calculated for each set of membranes corresponding to a concentration level. Table 5 summarises what has just been described.

### 4.3. Results Discussion

In the following, the sensitivity observations based on the previously defined figures of merit are quantified numerically. From the values reported in Table 5, it is evident that:

- The normalised impedance variation at 20 Hz becomes increasingly negative with rising pollutant concentrations;
- The spectral spread $s_{m}\left(t_{i}\right)$, particularly within the first 30 s following pollutant injection, increases with concentration, albeit in a moderate and non-linear fashion.

While the spread is less sensitive to variations at the lowest concentration (0.1 mM), it demonstrates pronounced differentiation for concentrations more than 1 mM.

Considering the standard deviation values and assuming Gaussian-distributed observations (coverage factor equal to 1 and a confidence level around 68.4%), the following conclusions can be drawn:

(i) Pollutant Detection—The variation at low frequencies is sufficient to detect the presence of a pollutant;
(ii) Concentration Discrimination (Low–Medium)—The combined use of low-frequency variation and spectral spread enables discrimination between 0.1 mM and 1 mM concentrations;
(iii) Concentration Discrimination (High)—The spread metric alone can distinguish concentrations approaching 10 mM.

These findings confirm the potential of the proposed system to act not only as a sensitive pollutant detector (detection limit around 0.1 mM) but also as a selective sensor capable of resolving distinct concentration levels (selectivity threshold around 1 mM and above). While the primary objective of the present work was to develop a functional detection platform, the emergent selectivity capabilities constitute a promising avenue for future development and optimisation.

## 5. Experimental Comparison with a Commercial Instrument

To validate the performance of the system proposed in this study, despite its current prototype development status, this section presents an experimental comparison with a commercially available pollutant detection device. Despite their different basic operating principles, the objective is to assess the operational behaviour and performance similarity between the proposed system and a commercial benchmark. The commercial instrument used, the comparative measurement setup, the experimental procedures conducted, and the results obtained are described below. As in the previous section, qualitative and quantitative evaluations are carried out based on the previously defined performance metrics.

### 5.1. Commercial System Configuration and Test Description

**Figure 14:**
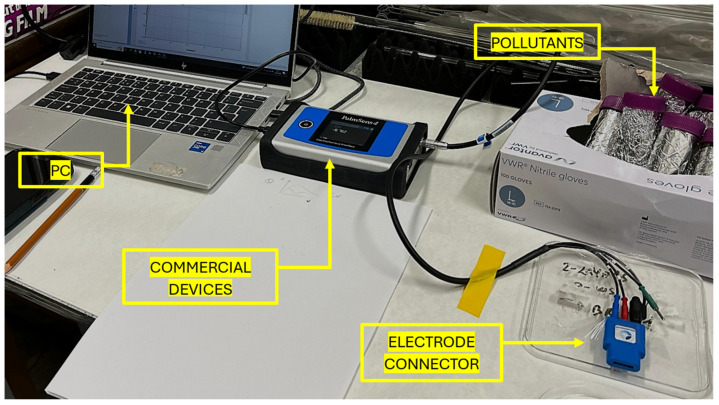
Experimental setup used for the comparison. It shows the commercial system used PalmSens4 (PalmSens, Houten, The Netherlands).

For this comparative analysis, the commercial device selected is the PalmSens4^TM^ instrument [49] (PalmSens, Houten, The Netherlands), used in conjunction with the ItalSens Carbon screen-printed electrode sensor (PalmSens, Houten, The Netherlands), as documented on the manufacturer’s official website [50]. The experimental setup adopted for this evaluation is depicted in Figure 14.

PalmSens4^TM^ (PalmSens, Houten, The Netherlands) is a versatile electrochemical instrument capable of executing various analyses, including EIS, which is critical for this comparison. The associated sensor consists of a three-electrode configuration: a working electrode with an active area of 0.07 cm^2^, a counter electrode, both made of carbon, and a silver-based reference electrode. To ensure a valid and meaningful comparison, the experimental protocol previously applied to the nanomembrane-based system was replicated here, fully aligned with the hardware and software constraints of the commercial platform.

Impedance measurements were conducted across the same frequency range as in the prior tests, using an excitation signal composed of a 1 V DC offset and a 10 mV AC component, for a total duration of 20 min per test. The DC component is necessary for this type of electrode. Unlike the proposed system, which continuously measures over time, the commercial instrument requires approximately one minute to complete a measurement. During the first five minutes, measurements were taken without the analyte to establish a reference. Subsequently, the same pollutant solution described previously was introduced. Identical BQ concentrations were tested, and each concentration was evaluated using four independent sensors, totalling 16 sensors overall. These procedures were adopted to ensure consistency and comparability across both platforms.

### 5.2. Results Discussion

In contrast to the approach adopted for the nanomembrane-based system, the impedance data acquired during the initial five minutes (i.e., before pollutant exposure) were excluded from the analysis. This exclusion is necessary due to the behaviour of the commercial electrode when operating in air, where it exhibits characteristics of an open circuit. Consequently, normalisation to a pre-drop baseline, as applied in the proposed system, is not feasible. Therefore, all data are interpreted in absolute terms.

**Figure 15:**
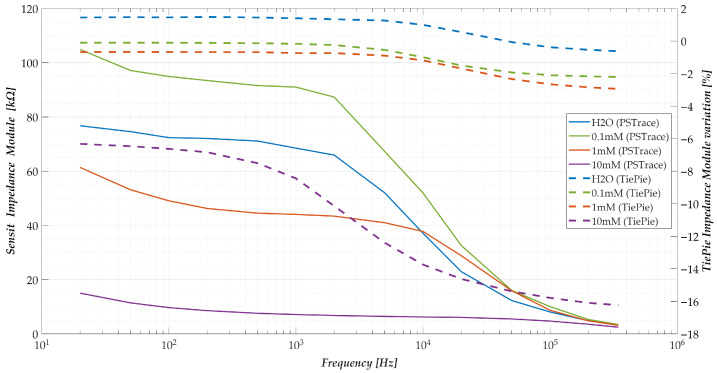
Frequency response relative to the impedance modulus offered by the commercial system used, at 180 s after the pollutant drop, for each concentration tested (solid lines—left axis). Show the comparison with the trend obtained, in terms of change in impedance modulus, at the same instant, with the method proposed in the work (dashed lines—right axis).

Figure 15 presents the impedance magnitude spectra acquired 180 s after pollutant introduction for each tested concentration, using one representative electrode per concentration. For comparative purposes, the corresponding impedance variations obtained using the proposed system simultaneously are also included in the figure, plotted on a secondary vertical axis (right) and expressed as relative changes from the baseline. This dual representation enables a direct visual comparison of the qualitative behaviour exhibited by both systems.

Both systems demonstrate similar frequency-dependent trends, characterised by a decrease in impedance magnitude at higher frequencies. However, a notable discrepancy emerges in terms of absolute impedance levels: while the proposed system operates within the low hundreds of ohms (as shown in Figure 12)—facilitating straightforward acquisition and analysis—the commercial device operates within the tens to hundreds of kilo-ohms range, which presents greater challenges in terms of signal handling and instrumentation. This difference stems from the inherent material and structural distinctions between the nanomembrane-based electrodes and the commercial carbon-based electrodes.

Furthermore, disparities in resolution and sensitivity are also evident. The commercial system demonstrates limited capability in reliably distinguishing between 0 mM and 0.1 mM concentrations, as indicated by overlapping responses. In contrast, the proposed system displays a clear monotonic relationship between concentration and impedance variation, reinforcing its superior discriminative capability.

### 5.3. Quantitative Analysis of Results

To provide a more detailed performance evaluation of the commercial system, Table 6 reports the average impedance magnitude at the lowest analysed frequency (20 Hz), along with the corresponding frequency spread as previously defined, both expressed in absolute terms. Standard deviations are included to account for sensor-to-sensor variability. For each tested concentration, the metrics are presented at three distinct time points: 60 s, 120 s and 180 s post-pollutant exposure.

The values reported in Table 6 corroborate the qualitative insights drawn from Figure 15. Specifically, measurements at 0 mM and 0.1 mM are statistically indistinguishable when considering the standard deviation, indicating an insufficient resolution for reliable detection of low-level concentrations. A general decreasing trend in the 20 Hz impedance magnitude is observed with increasing concentration, except for the 0.1 mM case. Moreover, analysis of the frequency spread in the commercial system yields limited additional insights into pollutant concentration levels, suggesting a lower sensitivity compared to the proposed platform.

## 6. Conclusions

This work presents the development and validation of a portable, low-cost and real-time measurement system for detecting waterborne pollutants, employing nanostructured PPF+Ni membranes as sensing elements. The proposed system directly addresses the critical need for decentralised and accessible monitoring solutions, in alignment with the objectives outlined in the United Nations 2030 Agenda for Sustainable Development. Building upon the authors’ prior work on the material-level sensing capabilities of nanomembranes, the focus of this study has shifted toward system-level design and experimental validation of a fully functional, field-deployable prototype.

The methodology is based on EIS, selected for its sensitivity to interfacial phenomena induced by pollutant adsorption. The proposed system features a custom-designed impedance measurement architecture, leveraging the voltage divider principle to optimise and simplify signal acquisition (avoiding current measurement). This approach enables accurate impedance readings while maintaining circuit simplicity, scalability and compatibility with microcontroller-based platforms. A key strength of the system lies in its configurability: by adjusting the impedance of the reference sample, it can adapt to a wide range of sensor types of different natures, thus improving adaptability and scalability. Moreover, the system’s robustness to environmental variations such as temperature and humidity was experimentally verified, confirming the stability of its operation under realistic usage conditions.

Experimental validation was performed through controlled exposure to BQ, which was used as a reference pollutant at increasing concentrations. The proposed system demonstrated the ability to detect BQ concentrations as low as 0.1 mM, with a consistent monotonic trend in the impedance magnitude across tested frequencies and concentrations. Selectivity was particularly evident in the lower concentration range (0–1 mM), where the system achieved a distinguishable response. Specifically, impedance variations at 20 Hz showed significant discriminatory ability, with consistent response curves across multiple sensors, confirming both the sensitivity and repeatability of the measurement.

A comparative analysis with the PalmSens4^TM^ commercial electrochemical analyser—using ItalSens Carbon screen-printed electrodes and the same test conditions—further validated the effectiveness of the proposed system. While the commercial device exhibited high absolute impedance values in the tens to hundreds of kΩ range, the proposed platform operated effectively within hundreds of ohms, facilitating signal acquisition and simplifying instrumentation. Notably, the commercial system struggled to differentiate between 0 mM and 0.1 mM reliably, whereas the proposed system retained clear concentration-dependent trends. These results underline the enhanced resolution and application-specific sensitivity offered by the nanomembrane-based sensing approach. In future developments, a dedicated analysis will also be conducted on the impact of uncertainty and error propagation on the performance metrics discussed to quantify further the robustness and reliability of the pro-posed measurement architecture.

The demonstrated performance of the present platform warrants further development. In the next phase, selectivity and robustness will be rigorously assessed on real samples to quantify matrix effects, cross-analyte interference, and long-term stability under realistic operating conditions. To this end, future works will be dedicated into the integration of additional nanostructured materials and surface functionalisation to extend the detectable pollutant spectrum. These efforts are expressly aimed at translating the current laboratory validation into a solution capable of real-time and in situ monitoring.

In conclusion, the objective of designing and validating a portable, low-cost, and selective system for pollutant detection has been successfully achieved. The ability to perform real-time measurements with high resolution at low concentrations, coupled with flexibility in sensor interfacing, positions this system as a promising tool for scalable environmental monitoring applications. 

## Figures and Tables

**Table 1:** Operating measurement specifications for the setup implemented. It shows the range and number of frequencies of the sinusoidal signals generated by the Tiepie HS5 for the EIS; while for acquisition using the TiePie HS6, it shows the sampling frequency, observation time and channel configuration in terms of full scale, number of bits and mode.

Parameter	Value
Input signal Frequency range	20 Hz ÷ 1 MHz
Number of frequencies generated	15 (logaritdmic step)
Sampling Frequency	50 × Frequency Input
Observation Time	Signal Period × 5
Voltage Full Scale	Auto-scale
ADC Resolution	14 bits
Channel modes	Differential

**Table 2:** Measurement performance in estimating the modulus of the impedance: average ($\bar{E}$) and maximum ($E_{M}$) errors at all the considered test voltages ($V_{S}$) and impedance values.

${\dot{Z}}_{X}$	$V_{S}=0.5$ V		$V_{S}=1$ V		$V_{S}=1.5$ V		$V_{S}=2$ V	
	$\bar{E}$	$E_{M}$	$\bar{E}$	$E_{M}$	$\bar{E}$	$E_{M}$	$\bar{E}$	$E_{M}$
[Ω]	[%]							
50	−0.015	0.85	0.21	1.2	0.039	1.3	0.017	1.2
100	0.080	1.3	0.24	1.7	0.081	1.8	0.092	1.7
200	0.60	1.5	0.81	1.8	0.75	2.0	0.78	2.1
500	1.5	3.5	1.5	3.7	1.9	4.1	1.9	4.2

**Table 3:** Measurement performance in estimating the phase of the impedance: average ($\bar{E}$) and maximum ($E_{M}$) errors at all the considered test voltages ($V_{S}$) and impedance values.

${\dot{Z}}_{X}$	$V_{S}=0.5$ V		$V_{S}=1$ V		$V_{S}=1.5$ V		$V_{S}=2$ V	
	$\bar{E}$	$E_{M}$	$\bar{E}$	$E_{M}$	$\bar{E}$	$E_{M}$	$\bar{E}$	$E_{M}$
[Ω]	[deg]							
50	−0.29	5.5	−0.29	4.9	−0.31	5.0	−0.32	5.1
100	0.31	6.6	0.35	6.1	0.33	6.2	0.34	6.3
200	−0.38	6.7	−0.36	6.2	−0.40	6.3	−0.41	6.3
500	1.6	11	1.5	11	1.5	11	1.5	11

**Table 4:** Identifier (M#) of the nanomembranes used for each tested pollutant concentration.

Index	Pollutant Concentration			
	0 mM	0.1 mM	1 mM	10 mM
**M#**	1, 2, 3, 4	5, 6, 7, 8	9, 10, 11, 12	13, 14, 15, 16

**Table 5:** Response $R_{m}\left(f_{i},t_{i}\right)$ obtained from the system regarding the amplitude of the minimum analysed frequency (20 Hz) and the amplitude value of the spread $s_{m}\left(t_{i}\right)$. It shows the mean and standard deviation values, in percent [%], at specific time instants after the drop. A colour scale has been added to facilitate reading.

Test	Time [s]	Value at 20 Hz [%]		Spread [%]	
		Average	DevSth	Average	DevSth
**0 mM**	30	0.36	0.10	2.98	0.28
	60	0.78	0.36	2.72	0.54
	90	0.95	0.38	2.75	0.63
	120	1.03	0.39	2.71	0.53
	150	1.16	0.26	2.78	0.64
	180	1.20	0.28	2.74	0.63
**0.1 mM**	30	−0.06	0.19	1.76	0.36
	60	−0.12	0.22	2.72	0.82
	90	−0.15	0.15	3.0	1.20
	120	−0.19	0.17	3.0	1.20
	150	−0.20	0.17	2.9	1.10
	180	−0.21	0.17	2.9	1.20
**1 mM**	30	−0.62	0.67	2.13	0.36
	60	−0.67	0.70	2.47	0.42
	90	−0.98	0.81	2.56	0.39
	120	−1.06	0.82	2.60	0.40
	150	−1.12	0.82	2.60	0.40
	180	−1.22	0.89	2.64	0.38
**10 mM**	30	−2.6	2.7	8.9	5.6
	60	−2.8	2.8	12.1	2.5
	90	−2.9	2.8	12.6	2.7
	120	−3.0	2.8	12.6	2.5
	150	−3.0	2.8	12.3	2.5
	180	−3.1	2.8	12.3	2.4

**Table 6:** Response $R_{m}\left(f,t\right)$ at minimum analysed frequency (20 Hz) and spread $s_{m}\left(t\right)$, both in terms of impedance module, obtained from the commercial system. It shows for both the mean and standard deviation values at specific time instants after the drop. A colour scale has been added to facilitate reading.

Test	Time [s]	Value at 20 Hz [kΩ]		Spread [kΩ]	
		Average	DevSth	Average	DevSth
**0 mM**	60	132.92	10.81	132.25	7.68
	120	118.70	14.23	118.07	10.07
	180	107.95	13.62	108.73	9.83
**0.1 mM**	60	137.04	4.45	136.26	3.15
	120	126.40	4.62	125.73	3.26
	180	120.08	7.08	119.3	5.00
**1 mM**	60	64.49	2.84	63.70	2.02
	120	61.01	2.65	60.24	1.86
	180	58.94	3.04	58.26	2.10
**10 mM**	60	19.2	1.20	18.5	0.90
	120	18.9	1.40	18.3	1.00
	180	18.7	1.50	18.1	1.10

## Data Availability

The data presented in this study are available on request from the corresponding author. The data are not publicly available due to their use in ongoing projects.

## Abbreviations

The following abbreviations are used in this manuscript:

EIS	Electrochemical Impedance Spectroscopy
PPF	Pyrolysed Photoresist Films
ADC	Analogue-to-Digital Converter
BQ	Benzoquinone
SEM	Scanning Electron Microscopy
PCB	Printed Circuit Board
